# Multiplex Immunofluorescence and the Digital Image Analysis Workflow for Evaluation of the Tumor Immune Environment in Translational Research

**DOI:** 10.3389/fonc.2022.889886

**Published:** 2022-06-27

**Authors:** Frank Rojas, Sharia Hernandez, Rossana Lazcano, Caddie Laberiano-Fernandez, Edwin Roger Parra

**Affiliations:** Department of Translational Molecular Pathology, The University of Texas MD Anderson Cancer Center, Houston, TX, United States

**Keywords:** immunotherapy, multiplex immunofluorescence, image analysis algorithms, tissue, machine learning

## Abstract

A robust understanding of the tumor immune environment has important implications for cancer diagnosis, prognosis, research, and immunotherapy. Traditionally, immunohistochemistry (IHC) has been regarded as the standard method for detecting proteins *in situ*, but this technique allows for the evaluation of only one cell marker per tissue sample at a time. However, multiplexed imaging technologies enable the multiparametric analysis of a tissue section at the same time. Also, through the curation of specific antibody panels, these technologies enable researchers to study the cell subpopulations within a single immunological cell group. Thus, multiplexed imaging gives investigators the opportunity to better understand tumor cells, immune cells, and the interactions between them. In the multiplexed imaging technology workflow, once the protocol for a tumor immune micro environment study has been defined, histological slides are digitized to produce high-resolution images in which regions of interest are selected for the interrogation of simultaneously expressed immunomarkers (including those co-expressed by the same cell) by using an image analysis software and algorithm. Most currently available image analysis software packages use similar machine learning approaches in which tissue segmentation first defines the different components that make up the regions of interest and cell segmentation, then defines the different parameters, such as the nucleus and cytoplasm, that the software must utilize to segment single cells. Image analysis tools have driven dramatic evolution in the field of digital pathology over the past several decades and provided the data necessary for translational research and the discovery of new therapeutic targets. The next step in the growth of digital pathology is optimization and standardization of the different tasks in cancer research, including image analysis algorithm creation, to increase the amount of data generated and their accuracy in a short time as described herein. The aim of this review is to describe this process, including an image analysis algorithm creation for multiplex immunofluorescence analysis, as an essential part of the optimization and standardization of the different processes in cancer research, to increase the amount of data generated and their accuracy in a short time.

## Introduction

The past several decades have seen growth in the use of cancer-screening protocols in the general population. These advances have increased the amount of material and work dedicated to cancer research and diagnosis. Cancer research and diagnosis use medical images such as mammography, magnetic resonance imaging, ultrasound, and microscopic tissue images (1), One of the most actively researched tasks in development is digital image analysis using computer-assisted diagnosis (2–6). Digital image analysis has become an essential part of cancer research, detection, treatment decisions, and surveillance routines, and it can be potentially used at many screening sites globally (7–10). Indeed, this technique has the much-needed potential to relieve pathologists’ workloads, diminish subjectivity, improvement of performance, and accuracy, allowing increased attention toward more challenging cases (1, 7, 10).

To this end, multiplexed imaging technologies have proven to be valuable techniques that enable the simultaneous detection of multiple markers in a single tissue section. Increasing demand for clinical trials evaluating large numbers of samples despite relatively limited amounts of tissue (especially brain tissue and other rare tumor tissue) available for research purposes has only increased the importance of these techniques (9). In cancer immunotherapy specifically, these tools have proven to be indispensable. For example, in numerous studies concerning programmed death-ligand 1 (PD-L1), multiplexed imaging technologies seemed to improve performance in predicting response to anti-PD-L1/programmed cell death protein 1 (PD-1) treatment of different solid tumors, as well as tumor mutational burden and gene expression profiling (11–13). Investigators have implemented and adopted multiplexed imaging technologies in research, and they will probably do so in clinical settings in the near future, enabling detailed cell structure, functional state, and cell-cell interaction analysis (14).

**Figure 1:**
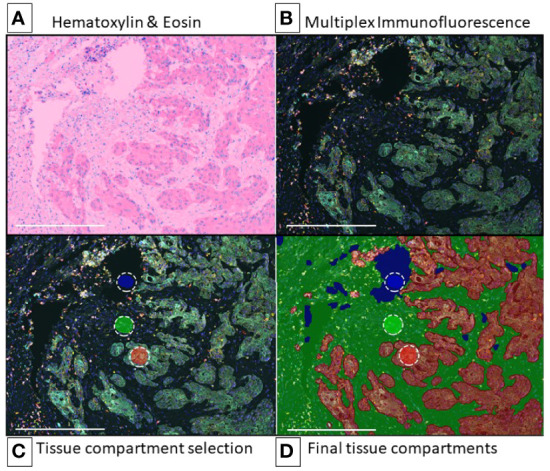
egion of Interest and Image Preparation for Multiplex Analysis Using Tyramide Signal Amplification. Representative digital image of non-small cell lung cancer for multiplex immunofluorescence phenotyping analysis (10x – scale bar: 500 μm). **(A)** Hematoxylin & Eosin (H&E) (representative H&E image view provided by the software during analysis). **(B)** Multiplex immunofluorescence image view for ROI (composite image). **(C)** Multiplex immunofluorescence image on intratumoral region with training regions for tissue segmentation, red (tumor), green (stroma), blue (glass). **(D)** Region on interest (intratumoral) after training algorithm for tissue segmentation, red (tumor), green (stroma), blue (glass). Composite image from inForm ^®^ image analysis software, Akoya bioscience. Scanner Vectra Polaris.

To take full advantage of multiplexed imaging technologies capabilities, such as telepathology, second opinions, education, and big data generation, cancer researchers have made many advances in histopathological whole slide image analysis (15). For example, improved digital image analysis software has enabled the compartmentalization of tissue into its components by selecting representative tissue compartment examples, such as tumor and stroma (**Figure 1**). In addition, lymphoid tissue and glass/background categories can be included in the tissue compartmentalization process; the former would be helpful for tissue morphometric analysis. Furthermore, detecting their expressed proteins through sophisticated algorithms generates large amounts of digital information that can be integrated with clinical information thanks to medical digitizing technologies (2, 15, 16). Histological analysis of tissue patterns by using computer-aided image processing to perform disease classification is made possible by significant developments in digitized histological studies (7–9, 15, 17). Similarly, quantitative pathological image characterization is essential for clinical applications, as it decreases interobserver and intra-observer variations and for research applications, particularly for understanding the development mechanisms and biology of cancer (7). Furthermore, quantitative analysis of immunohistochemically stained samples for immune cells or tumor biomarkers *via* digital pathological approaches increases the precision and accuracy in measurement of the expression of surface proteins in pretreated and treated samples to evaluate the tumor microenvironment (TME) composition and its modifications in response to immunotherapy or targeted therapy (18).

This review aims to explain how multiplexed imaging technologies are applied to translational research and tumor immune environment characterization. To achieve research objectives by using digital image analysis, optimizing and standardizing multiple immunofluorescence validation and panel design in this way and the different steps of the image analysis workflow, which is necessary to improve imaging data accuracy.

## Tumor Immune Environment

Cancer treatment has changed dramatically in the last two decades due to an increased understanding of tumor biology and its mechanisms of development, allowing the development of targeted therapy (19, 20). Immunotherapy enhances the body’s antitumor immune response by promoting tumor immune recognition, immune activation, and immune response persistence. As a result, it has become a valuable cancer treatment option for those suitable cancer patients (21–27). Since the development of immune checkpoint inhibitors (ICIs), the incidence of postoperative recurrence of cancer has decreased, and progression-free survival (PFS) and overall survival (OS) have improved (28–30). As an example, Nadim clinical trial (NCT03081689), a multicenter open-label clinical trial in which 46 patients with resectable non-small cell lung cancer (NSCLC) treated with neoadjuvant chemo-immunotherapy, were evaluated, it was reported a progression-free survival (PFS) of 36 months and overall survival (OS) of 42 months (30). Furthermore, a systematic review of the literature has also reported improvement in 12 months’ overall survival of cancer patients treated with immunotherapy, alone or in combination (31). However, although immune checkpoint inhibitors have demonstrated high efficacy in the treatment of tumors such as melanoma, non-small cell lung carcinoma, renal cell carcinoma, and Hodgkin lymphoma (32, 33), treatment-related adverse effects and toxicity are still an important issue in these patients, and more studies are needed (34, 35).

### Cancer Immunoediting

**Figure 2:**
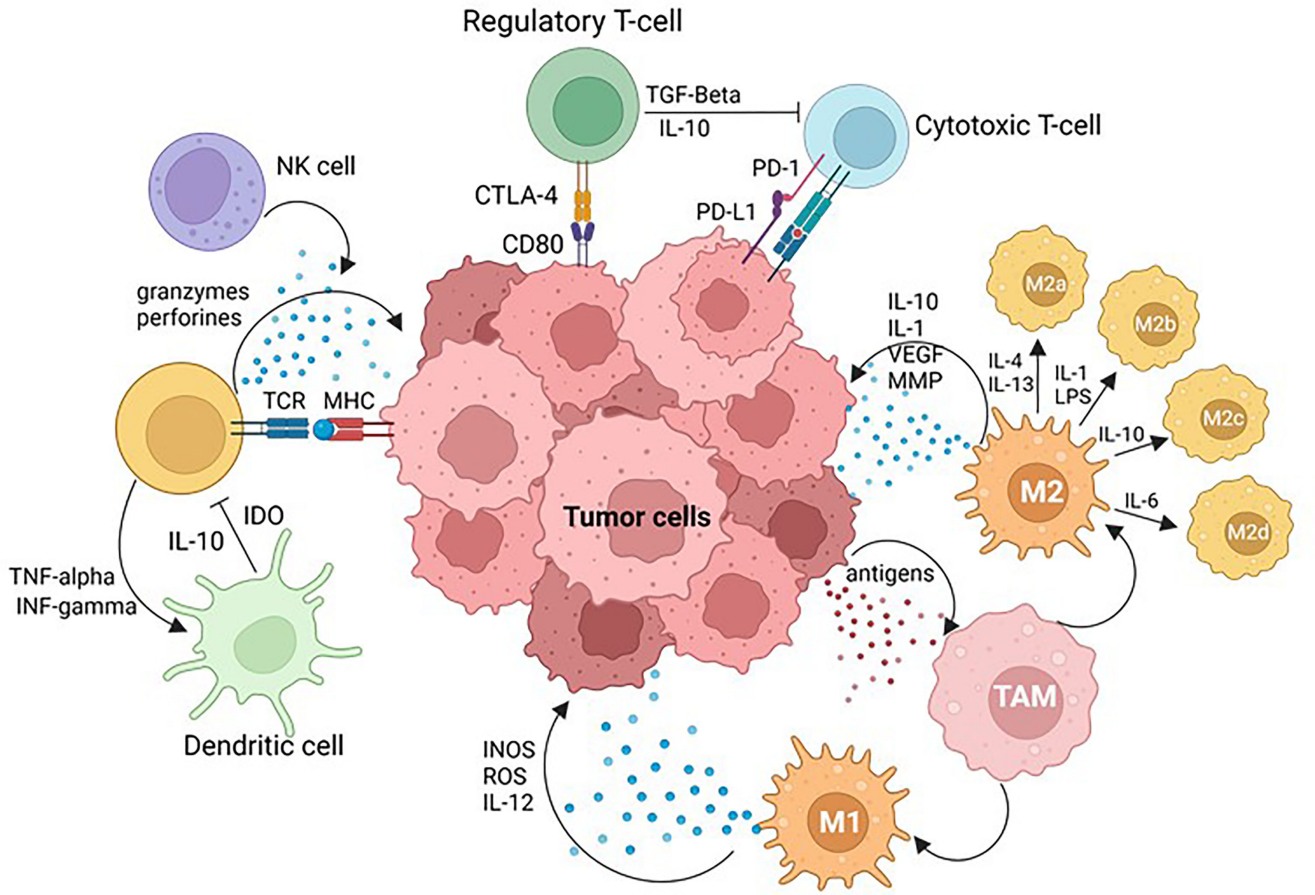
Tumor Microenvironment (TME). PD-L1 tumoral cell expression and PD-1 immune cell axis plays a key role in physiological immune homeostasis and contributes to tumor cell immune evasion. Cytotoxic T-lymphocyte antigen 4 (CTLA-4) ligation to CD28 quantitatively augments TCR-mediated signals, as well as activating independent pathways to upregulate CD28-mediated cytokine production and proliferation, raising the threshold needed for T-cell activation and arresting T-cell-cycle progression. Simultaneous recognition of a specific major histocompatibility complex (MHC)–peptide complex by the T-cell receptor (TCR) and of B7-1 (CD80) or B7-2 (CD86) also results in T-cell activation, cytokine production, proliferation and differentiation. Natural killer (NK) cells are effector lymphocytes that play protective roles against both infectious pathogens and cancer. Dendritic cells (DC) are professional antigen presenting cells, able to induce naïve T cell activation and effector differentiation, involved in the induction and maintenance of immune tolerance in homeostasis. Tumor associated macrophages (TAMs) are constituted by: M1 macrophages that play critical roles in innate host defense by producing reactive oxygen/nitrogen species (ROS/RNS) and pro-inflammatory cytokines such as IL-1β, IL-6, tumor necrosis factor α (TNF-α), and M2 macrophages that produce anti-inflammatory cytokines such as IL-10, IL-13 and TGF-β to promote tumor development. Image created with BioRender.com.

Tumor differentiation, epigenetics, tumor spread, and immune evasion are all influenced by the TME. The TME is very diverse and is constituted by several cell types and a wide variety of chemical molecules produced and released by tumor cells, stromal cells, and other cells (36) (**Figure 2**). Both innate and adaptive immunity have roles in immune surveillance, also known as cancer immunoediting, with three phases: activation, equilibrium, and escape (37). During the elimination phase, the host protective mechanisms of the immune system detect and attack the tumor cells that express stress-related molecules. Some of these molecules include lymphocytes’ host effector molecules such as interferon-gamma and perforin, targeting tumor cells and enhancing the cross-presentation between dendritic cells (DCs) CD103+ and CD8+ T cells, among other immune phenomena (37–40). Tumor cells arise when altered cells escape immune control during this phase; despite the immune system’s ability to recognize and kill tumor cells, a tumor can continue to grow (equilibrium phase) and eventually escape surveillance (escape phase) (37, 41).

### Antitumor and Pro-Tumor Immune Subsets

Antitumor immune responses are mediated by lymphocytes such as natural killer (NK) cells, CD8+ T cells, and CD4+ helper T (Th) cells, as well as proinflammatory (M1) macrophages and DCs. In contrast, pro-tumor immunity is mediated by a heterogeneous population of myeloid-derived suppressor cells (MDSCs) and Foxp3+ regulatory T cells (Tregs) (36). Tumor cells evade immune cells through various mechanisms, including inhibition or loss of tumor antigens, the release of immunosuppressive extracellular vesicles such as exosomes, the release of immunosuppressive molecules such as interleukin-10, and transforming growth factor (TGF). Other described mechanisms are shedding of soluble major histocompatibility complex I (MHC-I), loss of adhesion molecules such as intercellular adhesion molecule-1 (ICAM-1), development of apoptosis through tumor necrosis factor-related apoptosis-inducing ligand (TRAIL), resistance to apoptosis *via* upregulation of BCL-2, and induction of immune tolerance by the extensively used therapeutic target PD-L1 (36).

### Dendritic Cells

DCs are critical immune system regulators that also coordinate immune responses in tumors, even though other innate immune cells also play a role in immune surveillance and immune tolerance; for example, M1 macrophages neutralize tumors by secreting lytic enzymes, tumor necrosis factor-alpha, and oxygen and nitrogen reactive intermediates and by inducing antibody-dependent cell-mediated cytotoxicity (ADCC) (42, 43).

Tumor antigens are successfully engulfed and processed by immature DCs in the TME. However, DC maturation and activation are elicited by damage-associated molecular patterns, which cause DCs to lose their ability to collect antigens and gain the ability to offer these antigens to lymphocytes. Mature DCs move from the tumor surrounding tissue to lymph nodes, where they offer antigens to CD4+ and CD8+ T lymphocytes *via* major histocompatibility complex class II and I molecules, respectively (36). In some cases, despite its ability to mount antitumor immunity, the immune system is unable to limit the tumor’s growth. This tumor immune evasion is just as challenging to activate as immune activation. Therefore, a lack of appropriate DC stimulation is critical in the tumor immune environment. If the DCs in the TME are not fully matured, they will present tumor antigens in a tolerogenic manner, resulting in anergic/tolerant T cells. As juvenile DCs in the TME, regulatory and tolerogenic DCs (tDCs) exhibit low expression of co-stimulatory molecules like CD80 and CD86 but high expression of inhibitory molecules like PD-L1 and cytotoxic T-lymphocyte-associated antigen-protein 4 (CTLA-4) (44).

### Tumor Infiltrating Lymphocytes

Tumor-infiltrating lymphocytes are lymphocytes that infiltrate the tumor site as a result of molecular signals (41). These TILs are constituted by different T-cell subsets, such as regulatory T cells (Tregs) and innate lymphoid cells (ILCs), such as natural killer (NK) cells, among others. Effector T cells and natural killer cells are commonly elicited and attracted to the TME to kill cancer cells by recognizing tumor antigens and membrane ligands. In addition, immune cell infiltration, tumor cell growth, and metastasis are influenced by chemokines, selectins, and integrins secreted by tumor cells and their stroma, which also aid in the extravasation tumor-infiltrating lymphocytes into tissues (45).

## The Traditional Pathological Tissue Analysis

Pathologists have used hematoxylin and eosin staining to visualize multiple tissue components in microscopic examinations of biopsy and surgical samples for more than 100 years (7). In histopathological analysis, disease grading and diagnosis include recognizing tissue structures such as lymphocytes, cancer nuclei, and glands. The nature, degree, size, shape, and other morphological features of these structures are essential indicators of the presence and severity of the disease (46).

In traditional pathological slide analysis, optic microscopic assessment at different magnifications (e.g., 4x, 10x, 20x, 40x, 100x) provides different levels of information. High-power field microscopy provides information about cell shape, whereas low-power field microscopy provides structural understanding and enables the identification of complex architectural features and spatial relations (47). However, in digital image analysis, the quality, accuracy, and amount of information depend on the image resolution, field of view, and selected regions of interest when performing digital image analysis. In the same way, to identify target molecules expression with clinical utility and their semiquantitative intensity assessment in the tissue, IHC has been an essential auxiliary method for pathologists not only in the diagnostic routine but also in the research field, providing a high-performance assessment tool when compared with other biomarkers modalities (11, 48–50),

In digital image analysis, pyramidal image and whole-slide analysis can address the lack of architectural data when using high-power field magnification. In addition, because cellular and structural atypia are present in cancerous tissues, images of tumor sections obtained at different magnifications contain important data (47).

For example, when grading prostate cancer, some characteristics are associated with tumor aggressiveness, such as cell features, tissue architecture, and image complexity, which are important for determining the Gleason patterns. However, for some challenging cases, these histological features can be influenced by interobserver variability, and although M2 macrophages and regulatory T cells have been suggested as part of the immune-suppressive mechanisms that contribute to cancer progression (51), tumor immune environment characteristics in prostate cancer are still under investigation (52, 53). On the other hand, in breast cancer samples and tumor immune environment, has been described the dual role of the immune system in tumor development/progression and inhibition (54–56). In tumor immune environment studies of breast cancer patients, a complex dysfunction of T helper 2 cells associated with a low percentage of CD4+ and CD8+ cells has also been reported (57, 58). Furthermore, researchers have observed that large numbers of lymphocytes are highly predictive of a good outcome and improved survival in histopathological assessment (46, 54, 56).

Another assessment for breast cancer is the study of tumor infiltrating lymphocytes (TILs) as a strong prognostic biomarker of good outcome for early-stage triple negative breast cancer (TNBC) and HER2+ breast cancer, which has been included in several international guidelines such as St. Gallen consensus (59), the European society of medical oncology (ESMO) (60), and the World Health Organization (WHO), as a criteria for pathology assessment and reporting (61–63).

Due to the high relevance of the tumor immune environment and the dynamic interrelations among its components for developing novel therapeutic strategies, a high degree of inter-observer and intra-observer variability in tissue analysis is a challenge for pathologists. So digital image analysis in translational research for cancer immune environment represents a good opportunity to address this issue and also generate big data from the whole slide image analysis by using multiplexed technologies for phenotyping (17, 56, 58, 64, 65).

## Tumor Immune Microenvironment Research Approaches

**Figure 3:**
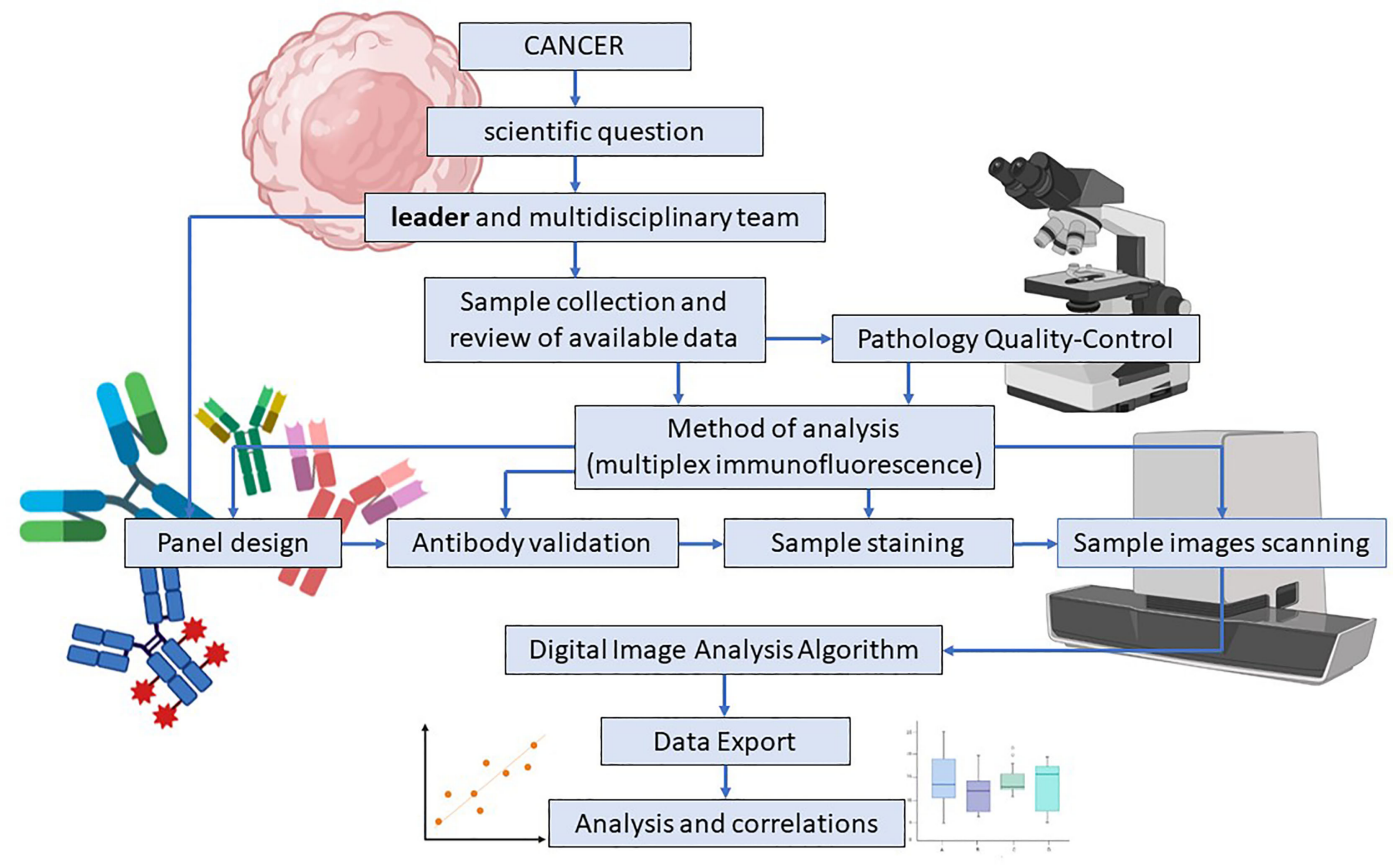
Study and multiplex immunofluorescence panel design in translational research. Multiplex Immunofluorescence (mIF) panel design for translational research starts with a scientific question and a multidisciplinary team guided by a scientific leader who addresses their skills and efforts to accomplish the research goal. Sample collection, data review, and pathologist quality control are simultaneously applied to determine the study’s feasibility. Antibody validation is an essential part of the process and assures that the expressed surface proteins and their intensity are correctly detected. High-resolution image scanning provides high-quality images for analysis purposes. Finally, when the image analysis algorithm is applied, data results are exported, analyzed, and correlated. Image created with BioRender.com.

In immuno-oncology research, understanding the complexity of the tumor immune microenvironment is essential. The study of the tumor immune environment is made possible by obtaining immune signature data using different techniques, such as flow cytometry, IHC, and multiplex immunofluorescence (mIF). In translational research, the panel design process requires the collaboration of a multidisciplinary team and is constituted by consecutive steps pursuing to find the answer to the scientific question (**Figure 3**).

Although flow cytometry has been used for immunophenotyping, this method requires cell disaggregation, which leads to loss of tissue architecture and information on spatial relationships between tumor and immune cells (66). IHC has also been used for immune cell phenotyping, and this technique preserves tissue architecture and the spatial relationship among cells. However, the amount of molecular proteins that can be detected simultaneously is limited (11, 50, 67). In the same way, the analysis of multiplexed images preserves the tissue architecture and the spatial relationships among all of the cell populations, which makes it a handy tool for characterizing the TME in translational research and obtaining reliable information (68–70). However, each technique offers a different perspective and data, so flow cytometry, IHC, and mIF must be considered complementary techniques and not excluded.

In addition, mIF can be used to evaluate protein localization in tissue samples using an immunofluorescent labeling-based image analysis algorithm (68). Also, given a reasonable number of markers in the same tissue section, it can facilitate the exploration of tissue morphology, spatial distributions, subcellular features, and cell surface proteins (71). Different technologies are capable of capturing the immune signatures of cells; in mIF, once different fluorochromes are attached to tissue proteins, signals can be extracted according to wavelength band from a series of scanned images, and the signals from various fluorophores are separated according to the signals created by Opal dyes (72, 73). To produce enzymatic signal amplification, tyramide molecules are conjugated with these fluorescent dyes (74).

An essential component of computerized image analysis in digital pathology is ascertaining prognostic markers’ function and expression patterns to predict cancer outcomes and survival (17, 64, 65). In this sense, immunotherapies have provided an increased understanding of patients’ TME and clinical response to treatment with immunotherapy. In addition, they have enabled clinicians to determine the appropriate indications for cancer treatment and identify possible effective drug combinations. For example, despite the efficacy of inhibitors of programmed cell death protein 1 (nivolumab and pembrolizumab) and PD-L1 (atezolizumab and durvalumab) in the treatment of melanoma and other solid tumors, physicians have observed resistance and relapse in other cancer patients with PD-L1 expression in malignant cells (75, 76). Similarly, investigators identified the simultaneous presence of cytotoxic CD8+ cells and expression of PD-L1 in tumors as a reliable prognostic marker for gastric cancer (77). From these findings, we can infer that due to the complexity and diversity of the tumor immune environment, more sophisticated techniques are required to identify and study different immune cell subsets and analyze cell-cell interactions in human tumor samples (78).

## Multiplex Immunoflourescence for Immune Cell Profiling

Several technical considerations must be addressed for developing a consistent mIF imaging platform, such as comprehensive tissue quality standards, a standardized multiplex assay staining scheme, the ability to quantify several markers in a specific region of interest, and experimental reproducibility both within and between laboratories (79).

### Antibodies for Multiplex Immunofluorescence

Because of their superior specificity, reliability, low frequency of staining background, and lot-to-lot variability, monoclonal antibodies are frequently used for immunohistochemical and immunofluorescent panel validation and analysis. Also, when compared to polyclonal antibodies, monoclonal antibodies are specific to target antigens, whereas polyclonal antibodies bind to various epitopes on the same protein and are acquired from experimental animals *via* repeated antigen stimulation (80).

### Tyramide Signal Amplification and Target Biomarkers

**Figure 4:**
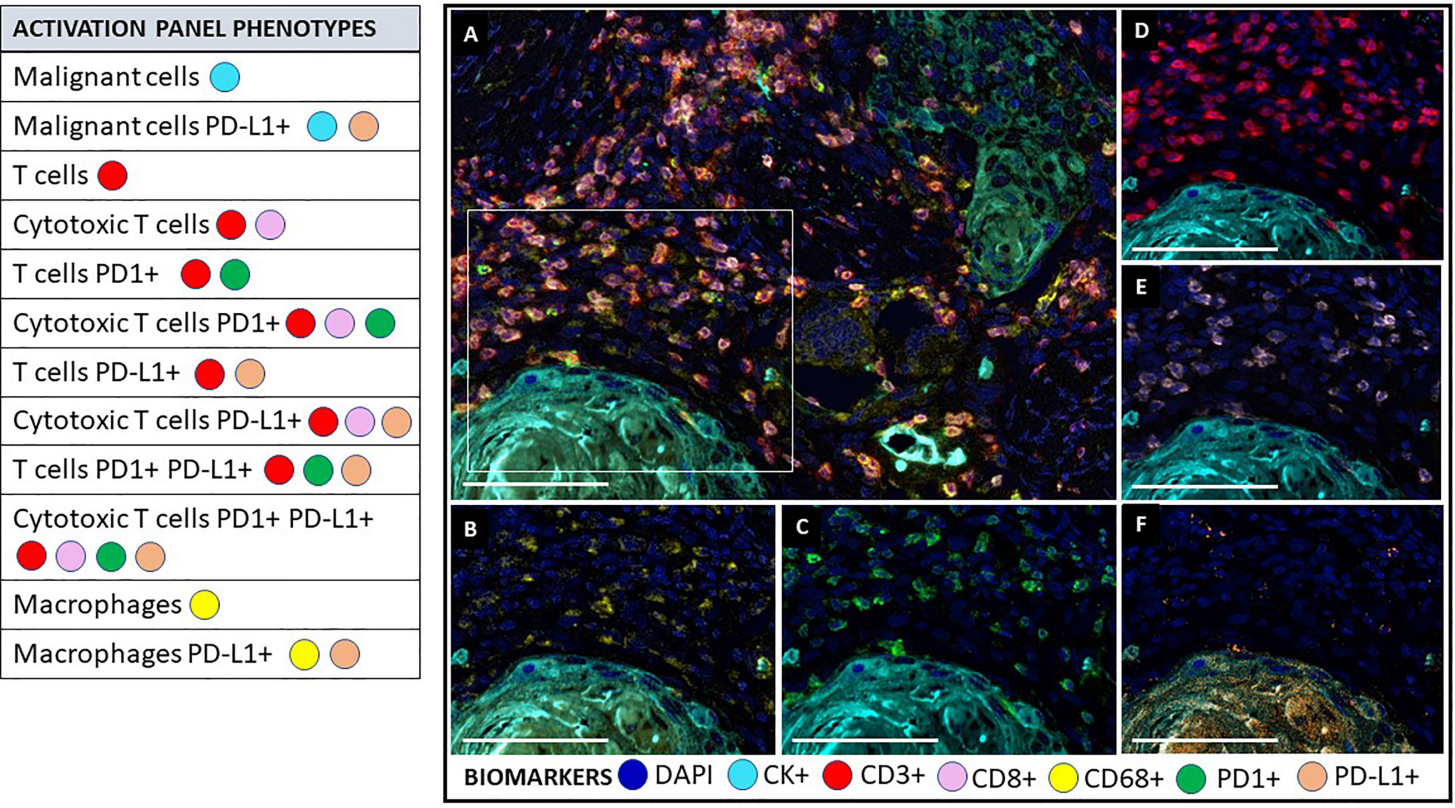
Multiplex immunofluorescence activation panel. The activation panel includes a universal biomarker for nuclear detection (DAPI) and cytokeratin for the detection of epithelial cells (malignant cells), which is also helpful for the compartmentalization of the image (tumor vs. stroma). TME and its different immune cell populations are detected using CD3+ for all lymphocytes and co-expressed CD8+ for cytotoxic T cells. Biomarker CD68+ identify macrophage population. This panel also includes biomarker expression for PD1+ and PDL1+ in immune cells and tumor cells to determine their activator or inhibitor status. **(A)** Composite image of TME from oral squamous cell carcinoma, showing all markers of a multiplex immunofluorescence “activation panel”, activated simultaneously. **(B)** Composite image showing CD68 positive expression (yellow) of immune cells in the stromal compartment. **(C)** Composite image showing PD1 positive expression (green) of immune cells in the stromal compartment. **(D)** Composite image showing CD3 positive expression (red) of immune cells in the stromal compartment. **(E)** Composite image showing CD8 positive expression (pink) of immune cells in the stromal compartment. **(F)** Composite image showing PD-L1 positive expression (orange) of immune cells in the stromal compartment. inForm ^®^ image analysis software, Akoya bioscience (scale bar: 100 μm). Scanner Vectra Polaris. DAPI (blue-DAPI), cytokeratin (cyan-opal 620), CD3+ (red-opal 690), CD8+ (pink-opal 540), CD68+ (yellow-opal 520), PD1+ (green-opal 650), PD-L1+ (orange-opal 570).

**Figure 5:**
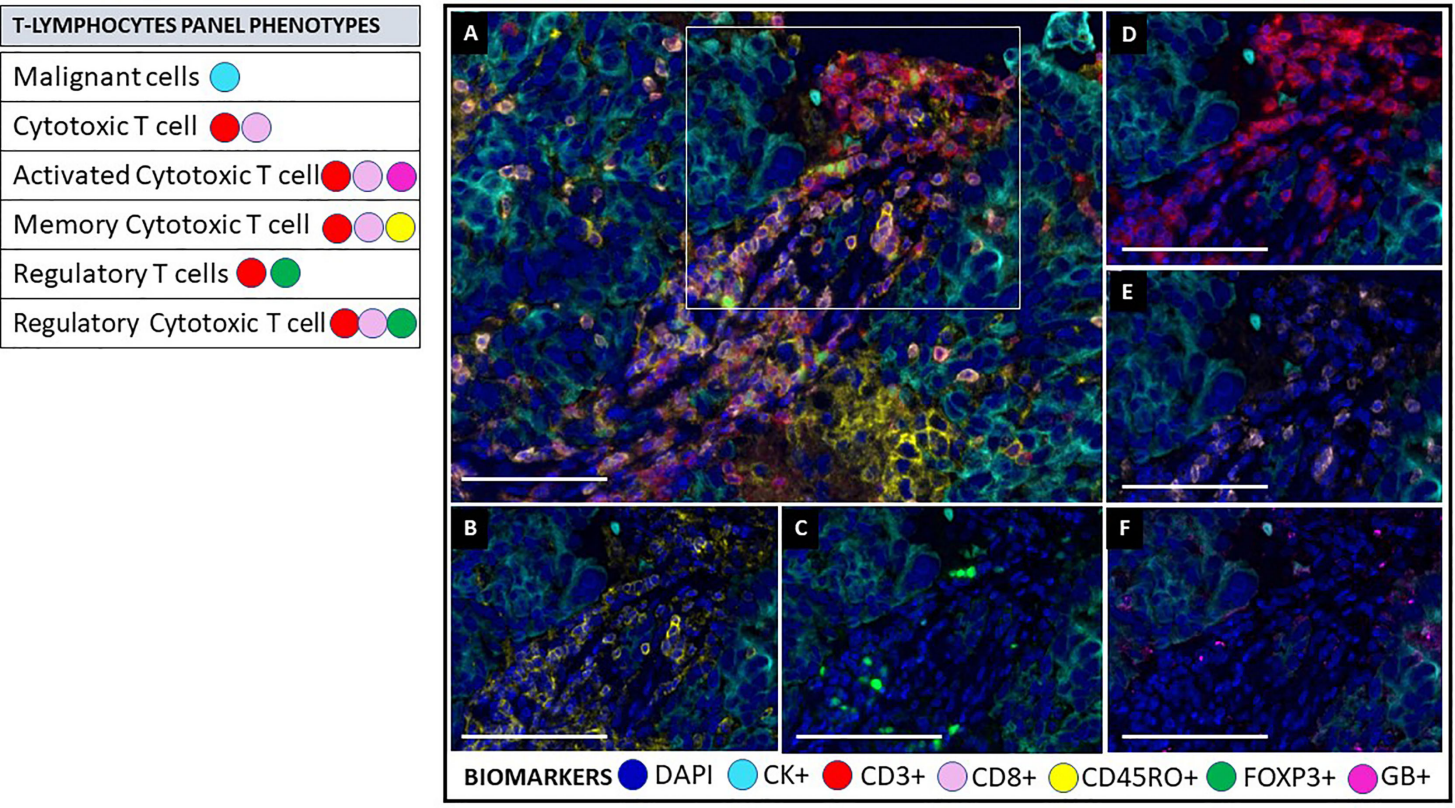
Multiplex immunofluorescence T lymphocytes panel. As on the activation panel, the T lymphocytes panel includes a universal biomarker for nuclear detection (DAPI) and cytokeratin for detection of epithelial cells (malignant cells), which is also helpful for compartmentalization of the image (tumor vs. stroma). T lymphocyte populations are detected using CD3+ for all lymphocytes, co-expressed CD8+ for cytotoxic T cells, and co-expressed FOXP3+ for regulatory T-cells. This panel includes granzyme B+ (GB) and CD45RO+ to identify activated T-cells and memory T-cells, respectively. **(A)** Composite image of TME from oral squamous cell carcinoma, showing all markers of a multiplex immunofluorescence “T lymphocytes phenotypes”, activated simultaneously. **(B)** Composite image showing CD45RO positive expression (yellow) of immune cells in the stromal compartment. **(C)** Composite image showing FOXP3 positive expression (green) of immune cells in the stromal compartment. **(D)** Composite image showing CD3 positive expression (red) of immune cells in the stromal compartment. **(E)** Composite image showing CD8 positive expression (pink) of immune cells in the stromal compartment. **(F)** Composite image showing GB positive expression (magenta) of immune cells in the stromal compartment. inForm ^®^ image analysis software, Akoya bioscience (scale bar: 100 μm). Scanner Vectra Polaris. DAPI (blue-DAPI), cytokeratin (cyan-opal 620), CD3+ (red-opal 690), CD8+ (pink-opal 540), CD45RO+ (yellow-opal 520), FOXP3+ (green-opal 650), Granzyme B+ (magenta-opal 570).

mIF tyramide signal amplification (TSA) combines staining with multispectral imaging analysis and allows for the creation of mIF panels of up to eight biomarkers, quantifies their expression, and characterizes their co-expression (cell phenotypes). A combination of biomarkers and individual fluorophores creates multispectral images that can generate several mIF panels for studying the tissue microenvironment (14, 80) (**Figures 4**, **5**). Targetable biomarkers like programmed cell death protein 1 PD-1/PD-L1 and their pathways can be analyzed to confirm the effect of immune treatments on the TME and their therapeutic benefit. Tumor immune environment analysis by mIF has been used to predict prognosis for diseases other than cancer and in the early phases of pathogenesis when signaling protein levels and functions are disrupted. Therefore, this technology (mIF) has played an important role in translational oncology research by increasing the understanding of the natural progress of the disease (81, 82).

Individual markers, such as those found in mIF panels, can be expressed in several types of cells simultaneously, and the detection threshold of the stained subcellular compartments, such as nuclei, cytoplasm, and cell membranes, must be addressed when creating the analysis algorithm. For example, tumor cells, macrophages, and lymphocytes can express PD-L1, but because lymphocytes are small cells with little cytoplasm that cannot always be differentiated from the cell membrane, they should be considered as positive for PD-L1 expression when cytoplasmic or membrane expression is present (83).

### Markers Co-Expression

Identifying the co-expression of several markers in specific cells is a strategy for confirming the specific cell target protein by including negative controls during the validation of the panel markers. In addition, including negative controls help eliminate autofluorescence interference during image preparation to ensure the acquisition of a clean signal for TME and mIF analysis (83).

For different mIF biomarker panels, including a common marker in the panels as an internal control is critical. For example, in immuno-oncology research, CD3 is commonly employed to analyze lymphocytes and their different subpopulations. In addition, although various tissue cut levels of formalin-fixed paraffin-embedded (FFPE) biopsy samples are utilized in the staining process, consecutive tissue cut levels should be used to obtain similar cellularity among panels during the staining procedure (83).

Cell phenotypes can be detected in any tissue compartment. They are defined by the co-expression of two or more surface markers simultaneously expressed in the known cell compartment during image analysis (83, 84) (**Table 1**).

**Table 1:** Examples of immune cells phenotypes according to their positive surface marker expression used in translational research.

Phenotypes	Surface markers (positive expression)
NK Cells	CD3/CD94/CD8
Cytotoxic Cells	CD3/CD8
T-Helper Cells	CD3/CD4
Memory Cells	CD3/CD45RO
Regulatory T Cells	CD3/CD4/FOXP3
Cytotoxic Memory Cells	CD3/CD8/CD45RO
T-Helper Memory Cells	CD3/CD4/CD45RO
Myeloid Cells	CD68/CD11c/CD11b/CD136/CD14/CD33
Lymphocyte LAG3	CD3/LAG3
Lymphocyte TIM3	CD3/TIM3
Lymphocyte OX40	CD3/OX40
Lymphocyte VISTA	CD3/VISTA
Macrophage M1	CD68/CD14
Macrophage M1 PDL1	CD68/CD14/PD-L1
Macrophage M2	CD68/CD163
Macrophage M2 PDL1	CD68/CD163/PD-L1
Macrophage M2 HLA-DR	CD68/CD163/HLA-DR
Cytotoxic Cell CD137	CD3/CD8/CD137
T-Helper activated ICOS	CD3/CD4/ICOS
Tumor Cells PD-L1	CK/PD-L1
Lymphocyte CD4+ PD-L1	CD3/CD4/PD-L1
Lymphocyte CD8+ PD-L1	CD3/CD8/PD-L1
Macrophage PD-L1	CD68/PD-L1
Cytotxic T cells PD-1	CD3/CD8/PD1
T-Helper Cells PD-1	CD3/CD4/PD-1
Cytotxic T cells CD137	CD3/CD8/CD137
T-Helper Cells CD137	CD3/CD4/CD137
Cytotxic T cells LAG3	CD3/CD8/LAG3
T-Helper Cells LAG3	CD3/CD4/LAG3
Cytotxic T cells TIM3	CD3/CD8/TIM3
T-Helper Cells TIM3	CD3/CD4/TIM3
NK Cells activated	CD94/GB/CD8
Cytotxic T cells GB	CD3/CD8/GB

### Tissue Processing and Effects on Multiplexed Images

Tissue processing and fixation are critical steps that must be standardized to avoid pre-analytical and analytical errors. Tissue ischemia and inappropriate tissue fixing can cause autolysis and degradation of proteins, RNA, DNA, and enzymes, decreasing antigenicity and antibody-binding site. Digital image analysis data from mIF can be negatively affected if extensive areas of necrosis, hemorrhage, and fatty tissue are present or a previous decalcification method was used (85). Some features must be considered regarding the tissue area selection, such as sample size, discordant diagnosis, amount of tumor cells, and selection of immune hot versus immune cold areas, among others (15, 85).

## Image Analysis Algorithm Creation

### General Perspectives

Tissue analysis of oncological pathologies by optic microscopy is made by manipulating the microscope’s magnification lens to gather information at the architectural and cellular levels. In machine learning-based digital image analysis, different images obtained at various magnification and quality resolution levels are often used (47, 86). Examination of whole slide images is the best approach to digital image analysis in histopathology because it yields reliable and accurate data and comprehensive information on spatial relationships among the different surface proteins expressed in the TME (87). However, since the amount of data from whole slide image analysis can be extensive and data analysis time consuming, if the experimental design allows, tissue microarray (TMA) can be used as an alternative technique that allows high throughput analysis of multiple tissue specimens at the same time, under experimental uniformity, amplifying scarce resources and decreasing volume assay, time and cost (88, 89).

Noteworthy, TME and tissue heterogeneity may vary throughout the whole tissue specimen. Therefore, the selected areas for analysis could be affected during the selection, so several studies have tried to apply AI algorithms under pathologist supervision to identify diagnostically important regions of interest (ROIs). These studies used image features such as pixels and textures, achieving tissue classification accuracy between 46% and 75% based on the quality of the tissue, quality of the image, and training data (90–92). These techniques must be selected according to the study objectives, and they should be considered complementary.

Many image analysis software packages are commercially available (**Table 2**), and they can be used for the phenotyping of samples through the input of specific tissue and cell features. In addition, open-source digital image analysis software packages are available, such as ImageJ (National Institutes of Health) and CellProfiler (**Table 3**). The analysis workflow of histopathological digital images carried out by using the different and currently available software packages are pretty similar. They all use reference points to recognize different structures that allow the software for unsupervised image exploration, image classification, image segmentation, and object tracking to identify surface proteins in mIF technology expressed in various tissue compartments (93).

**Table 2:** Representative and commercially available digital image analysis software packages.

Package	Manufacturer
inForm	Akoya Biosciences
HALO	Indica Labs
Visiopharm	Visiopharm
Aperio eSlide Manager	Leica Biosystems
CaseViewer	3DHISTECH
Augmentiqs	Augmentiqs
Image-Pro aiforia	Media Cybernetics Aiforia Technologies

**Table 3:** Representative open source digital image analysis software packages.

Package	Website
Fiji (ImageJ)	https://imagej.net/Fiji
QuPath	https://qupath.readthedocs.io/en/latest/
CellProfiler	https://cellprofiler.org/
ilastik	https://www.ilastik.org/
Orbit	http://www.orbit.bio/
Icy	http://icy.bioimageanalysis.org/
Cytomine	https://cytomine.com/

In developing an image analysis algorithm, machine learning and deep learning methods for hierarchal data clustering can be obtained by using the k-means method, which is a popular deep learning method, and density-based spatial clustering of application with noise (DBSCAN), another deep learning method that differs from the K-means on its less susceptibility for extremes median values (93–96).

The intensity of marker expression and morphology is essential for tissue classification, cell classification, and identification and measurement of protein expression. Then, the tumor sample images are analyzed using the principle of mutual identification and exclusion once the tissue characteristics are determined based on the immunological background regarding the tumor’s behavior reported by scientific literature and its relationship with the immune system according to the study’s objectives (97). Finally, a binary classification scheme is used to classify cells as positive or negative for a single marker. In this classification, the assessment of the surface markers is based on a cell attribute that is not shared by the rest of the cell population (78, 98).

**Figure 6:**
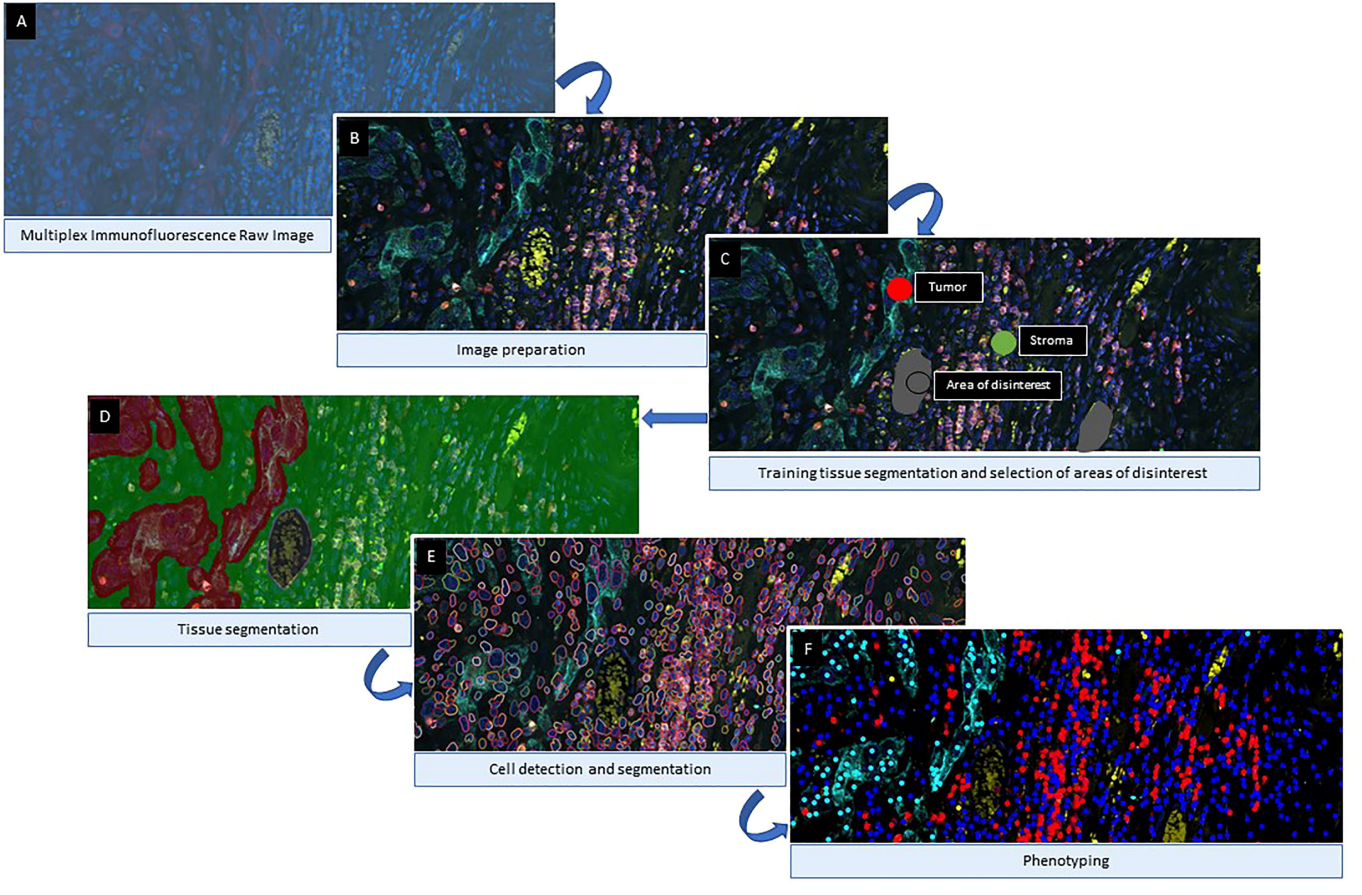
Algorithm Workflow of Multiplex immunofluorescence Digital Image Analysis. After image scanning, raw images **(A)** are prepared by activating the fluorochromes attached to the different cell surface proteins **(B)**. Then, for tissue compartmentalization, the software user can select the most representative examples of each compartment used during the training algorithm and exclude areas of disinterest **(C)**. Once the tissue compartment algorithm is applied and the areas are defined **(D)**, the next step is to define the cells limits **(E)** as individual objects to count for the analysis. The final part of the image analysis algorithm is the classification of the cells based on their phenotypes according to their surface protein expression **(F)**. Images examples from inForm ^®^ image analysis software. Akoya Biosciences.

When evaluating a large number of digital images, a combination of deep learning algorithms and machine learning is helpful in the analysis of mIF data and quantification of image features (97). For these algorithms to work effectively, they must recognize, learn from, and dynamically adjust to tissue heterogeneity. However, an algorithm may perform well for one image but poorly for another using fixed input parameters (99), making extracting information from digital slide images and analyzing it challenging. Once the images have been prepared by activating the fluorochrome detection for multiplexed digital image analysis, the following steps consist of tissue segmentation, cell segmentation, phenotyping, and data exportation (**Figure 6**).

### Tissue Segmentation

Image compartmentalization is one of the first steps in digital image analysis and algorithm creation. It demarcates a tissue sample’s compartments, such as the tumor, stroma, blood vessels, and other histomorphology areas. During this step, the software user must create and name the different tissue categories of interest and provide representative examples of them to achieve an accurate detection of the different tissue categories provided by the expert (**Figure 1**). The number of examples needed for each category may vary depending on the tissue heterogeneity and tissue preparation quality, staining quality, and image resolution. Usually, providing some of the best representative examples from the training areas is considered good practice, but the overall accuracy of tissue classification always depends on the quality of the training areas selected by the user and the software’s capabilities in interpreting those areas.

The user can also separate specific areas of disinterest, such as necrosis, tissue-processing artifacts, and staining artifacts. These areas can then be eliminated using the various methods embedded within a digital image analysis software program, such as manually drawing a perimeter around the object of disinterest or altering thresholds for lower or higher marker values. After doing so, the algorithm will disregard potentially confusing elements during its training process that may later put the algorithm’s accuracy at risk.

To quantify the total area of tissue to be analyzed, the user can create a new category representing the image areas that do not contain tissue. Some suggested names for this category are “glass” and “background” (94, 100). This helps the software save time when performing image analysis, as it can be configured during training to not apply the final image analysis algorithm to the areas without tissue.

### Cell Segmentation

Cell segmentation begins with nuclear detection. The digital image analysis algorithm combines different nuclear characteristics, the most important of which include size, roundness, edges, and texture, to identify the individual nuclei in a sample. Some tumor cells with high nuclear density may exhibit nuclear overlap, which may also be seen in neoplastic samples with high-density immune cell populations. However, this characteristic may be erroneously overlooked as a tissue artifact in either case. To address this nuclear overlapping issue, most digital image analysis software packages permit the regulation of nuclear segmentation aggressiveness. This function enables the splitting of a nuclear signal into the areas where the software user can identify more than one nucleus and thus prevents underestimation or overestimation of the cell population.

The cytoplasm and membrane are also considered critical features in each cell during cell segmentation, depending on the digital image analysis software program and its proper use.

Although a counterstaining nuclear marker (e.g., DAPI) should always be included in an mIF staining panel, a marker like cytokeratin, which is used for the identification of epithelial tumors, also can be employed as a cytoplasmic/membrane marker to improve segmentation of cells (85). In addition, the segmentation of immune cells also can be achieved by using membrane markers such as CD68 for macrophages and CD3 for T cells.

### Phenotyping, Single-Cell Marker Expression, and Cell Marker Co-Expression

**Figure 7:**
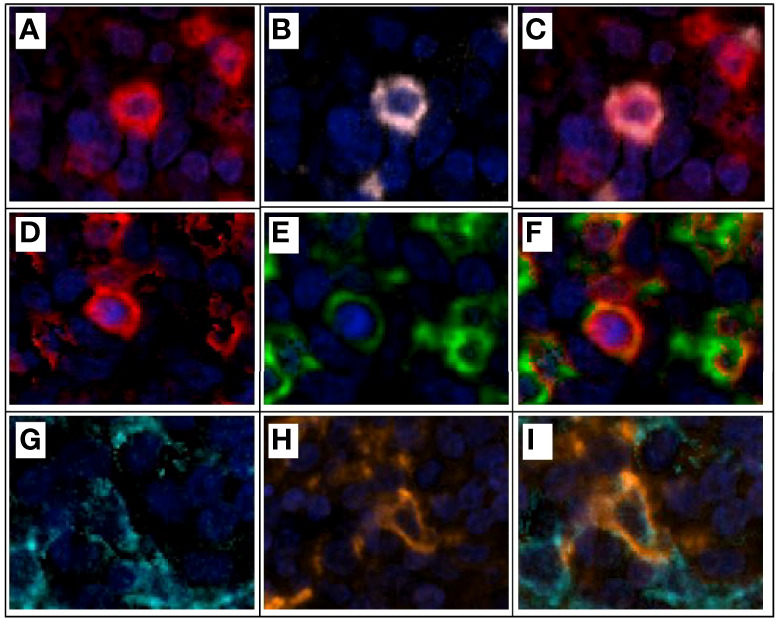
Multiplex immunofluorescence and colocalization examples. **(A)** CD3+ expression (red) on a lymphocyte T. **(B)** CD8+ expression (pink) of the same lymphocyte T in example **(A)**. **(C)** CD3+ and CD8+ expression and colocalization (red and pink) for a cytotoxic T lymphocyte. **(D)** CD3+ expression (red) on a lymphocyte T. **(E)** PD1+ expression (green) of the same lymphocyte T in example **(D)**. **(F)** CD3+ and PD1+ expression and colocalization (red and green) for a T lymphocyte PD1+. **(G)** Cytokeratin+ expression (cyan) of an epithelial tumor cell. **(H)** PDL1+ expression (orange) of the same epithelial tumor cell on example **(G)**. **(I)** Cytokeratin+ and PDL1+ expression and colocalization (cyan and orange) on an epithelial tumor cell expressing PDL1 on its surface for immune evasion. Composite image from inform ^®^ image analysis software, Akoya bioscience. Scanner Vectra Polaris.

An mIF marker panel can be analyzed using single-cell marker identification or according to the co-expression of two or more markers. The choice between these two approaches depends on the complexity of the markers in the panel, and the markers can be used as comprehensive or restrictive cell phenotype identifiers. In the single-cell marker approach, individual cell marker identification begins with creating an algorithm that identifies mutually exclusive markers in mIF panels, such as cytokeratin, CD3, and CD68. Then, the other markers are identified in consecutive steps in different phenotyping sessions. In the co-expression approach, co-expressed markers are identified by activating at least two different phenotypes simultaneously (**Figure 7**).

Using a visual approach, a pathologist or analyst familiar with the specific marker’s expression and location in the cells must correctly identify the marker or cell phenotype using the algorithm and avoid staining of the background and false positivity (85, 101). The co-expression of markers is accomplished by activating two or three markers simultaneously and verifying that they overlap in the correct pattern in the expected cells. However, using a control marker’s intensity when possible and adequate for the performance and training of the algorithm is recommended.

Digital image analysis for multiplex immunofluorescence can be performed in single or batch mode, both of which have advantages and disadvantages. Batch mode is faster and enables the analysis of a large group of samples in a shorter time than single-mode. However, due to the heterogeneity of the intrinsic tumor tissue and technical differences due to tissue processing, such as fixation and staining, applying a single algorithm in batch mode may be challenging, sometimes misreading tissue compartments and under/over detecting single nuclei. In addition, the application of a single digital image analysis algorithm requires selecting a training set group of images, which should be constituted by some of the most representative samples included in the study and show special characteristics that might present, such as mucin, cartilage, hemosiderin-laden macrophages, and others. Finally, to address the lack of accuracy during tissue and cell classification, the ideal software should allow real-time quality control supervised by a pathologist; whereas single-mode analysis can be more accurate than batch mode analysis, the former takes more time.

In addition, a training algorithm is applied to different images, and a pathologist must verify the accuracy of that application. Quality control in machine learning-based examination of biopsy samples, determination of the sample dimensions, and verification of accurate cell populations by a well-trained pathologist help ensure high-quality data from multiplex image analysis (85). Deep learning methods generate a significant amount of relational and non-relational data. This big-scale information requires automated or semi-automated storage, processing, and analysis, offering adequate and complete information that can be filtered and improved by automation and supervised under medical expert knowledge and allowing for finding data trends on a large scale to provide decision-making support (102, 103). However, handling all the generated data can be highly costly due to the different knowledge filed involved in the process, such as medical experts, technicians, data analysts, and technology development engineers, so much improvement must be done to expand the number of images and shorten their analysis results in a costless and timely manner.

## Translational Research, Digital Pathology Challenges, and Future Steps

Counting cells, blood vessels, and nuclei is a standard and necessary task in translational cancer research that can also be challenging due to interobserver variability. Thus, a primary objective of artificial intelligence-based tissue analysis is the promotion of reliability of the data obtained. For example, in comparing automated and manual object counts, authors have reported interobserver variations, finding an error range of 1.1-4.7% in the automated number of cell counts compared with manual counts (104). Similarly, in comparing manual and automated cell counts of immunohistochemically stained bone marrow biopsy samples, researchers observed a 2.8% to 10.0% discrepancy in these cell counts (105). Furthermore, the authors reported an error range of 19-42% between manual and automated mitosis counts in breast tissue samples visualized with Feulgen stain (106). These findings demonstrate that tissue analysis by pathologists can be subjective and prone to inter-observer and intra-observer variations (107). Therefore, improving the performance of object counts by using artificial intelligence is important to obtain more accurate and reproducible data on tissue samples.

Another challenge in mIF and digital image analysis is using different analytical approaches that are subject to potential errors that may affect the data output. For example, the selection of regions of interest in platforms such as mIF may be subjective, affecting the quality of the image analysis results. Therefore, whole slide images should be analyzed when possible to reduce variability in data results. Standardization of the different steps involved in any mIF project design, from panel antibody validation to the image analysis workflow, is necessary to overcome mIF and digital image analysis variations among researchers (15, 85).

**Figure 8:**
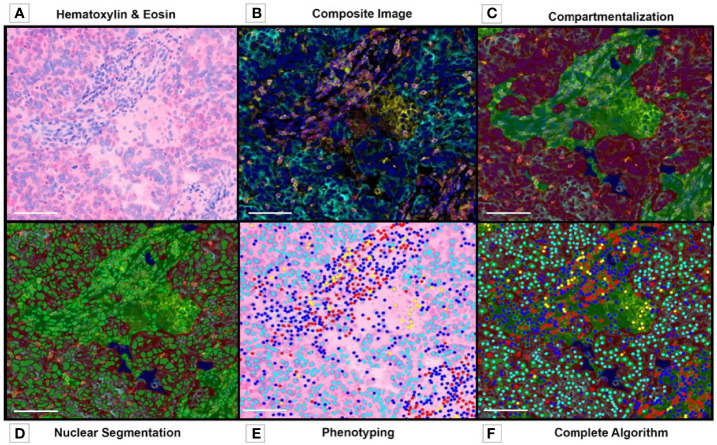
Multiplex Immunofluorescence. Image Analysis Overview. **(A)** Tumor and Stroma H&E image before preparation with multiplex fluorochromes. **(B)** Composite image, before algorithm analysis. **(C)** Tissue compartmentalization, tumor (red), stroma (green). **(D)** Nuclear detection and nuclear segmentation in both compartments (tumor and stroma). **(E)** Phenotyping map. **(F)** Complete algorithm visualization, including tissue segmentation, nuclear detection, cell segmentation and phenotyping. Composite image from inForm ^®^ image analysis software, Akoya bioscience (scale bar: 100 μm). Scanner Vectra Polaris.

The pathology community has been debating different assays to validate digital pathology in translational research, with little agreement on the parameters that should be used or how they could be used. We consider tissue quality, antibody validation, panel design, image resolution, and algorithm creation among the essential parameters for immuno-oncology and digital image analysis validation and the standardization of the analysis workflow to obtain reliable and reproducible results data (**Figure 8**).

Many new platforms, such as multiplexed ion beam imaging (MIBI), cytometry by time-of-flight, and CO-Detection by indEXing, are currently being used to perform high-plex immunoprofiling and cell phenotyping. However, all these platforms have in common the use of image analysis algorithms to manage large quantities of data. Thus, in the very near future, more studies will be needed for digital pathology validation and the establishment of a consensus regarding the assessment of results from all these platforms and the ones to come.

## Conclusions

Understanding the tumor immune microenvironment is important for cancer diagnosis and treatment. Thanks to the latest technologies in histopathology and digital image analysis, the development of tools such as mIF that allow for the focused study of proteins and cell surface markers that may be considered therapeutic targets is possible. In addition, significant advances in the acquisition, storage, and processing of digital images have enabled detailed studies of large quantities of material in a small amount of time, thus guaranteeing the reproducibility of results and their application to the development of new cancer treatments. Therefore, the design, validation, and standardization of an mIF marker panel offer an answer about the TME in the translational immuno-oncology field, and the creation of algorithms for the digital analysis of histological images with the help of available software packages are fundamental parts of understanding the tumor biology and immunology. Furthermore, applying these technologies logically and systematically facilitates the processing of large samples and images that can generate valuable and helpful information for developing new immunotherapies for cancer.

## Author Contributions

FR and EP conceived the idea and theme developed in this article and wrote most of the manuscript. SH, RL, and CL contributed their expertise to digital image analysis and immune profiling. EP developed the mIF technology in our laboratory and edited the manuscript. All authors approved the final manuscript.

## Funding

This manuscript was supported in part by the scientific and financial backing of the Cancer Immune Monitoring and Analysis Centers and Cancer Immunologic Data Commons Network provided through NCI Cooperative Agreement U24CA224285 for the MD Anderson Cancer Immune Monitoring and Analysis Centers and Translational Molecular Pathology Immunoprofiling Laboratory, the NIH/NCI under award number P30CA016672 and used the Institutional Tissue Bank, NIH SPORE grant 5P50CA070907-18, and Cancer Prevention & Research Institute of Texas grant MIRA-RP160688.

## Conflict of Interest

The authors declare that the research was conducted in the absence of any commercial or financial relationships that could be construed as a potential conflict of interest.

## Publisher’s Note

All claims expressed in this article are solely those of the authors and do not necessarily represent those of their affiliated organizations, or those of the publisher, the editors and the reviewers. Any product that may be evaluated in this article, or claim that may be made by its manufacturer, is not guaranteed or endorsed by the publisher.
